# Nondense mammographic area and risk of breast cancer

**DOI:** 10.1186/bcr3041

**Published:** 2011-10-21

**Authors:** Andreas Pettersson, Susan E Hankinson, Walter C Willett, Pagona Lagiou, Dimitrios Trichopoulos, Rulla M Tamimi

**Affiliations:** 1 Channing Laboratory, Department of Medicine, Brigham and Women's Hospital and Harvard Medical School, 181 Longwood Avenue, Boston, MA 02115, USA; 2 Department of Epidemiology, Harvard School of Public Health, 677 Huntington Avenue, Boston, MA 02115, USA; 3 Department of Nutrition, Harvard School of Public Health, 677 Huntington Avenue, Boston, MA 02115, USA; 4 Department of Hygiene, Epidemiology and Medical Statistics, University of Athens Medical School, 75 Mikras Asias Street, 115 27 Goudi Athens, Greece

## Abstract

**Introduction:**

The mechanisms underlying the strong association between percentage dense area on a mammogram and the risk of breast cancer are unknown. We investigated separately the absolute dense area and the absolute nondense area on mammograms in relation to breast cancer risk.

**Methods:**

We conducted a nested case-control study on prediagnostic mammographic density measurements and risk of breast cancer in the Nurses' Health Study and the Nurses' Health Study II. Premenopausal mammograms were available from 464 cases and 998 controls, and postmenopausal mammograms were available from 960 cases and 1,662 controls. We used a computer-assisted thresholding technique to measure mammographic density, and we used unconditional logistic regression to calculate OR and 95% CI data.

**Results:**

Higher absolute dense area was associated with a greater risk of breast cancer among premenopausal women (OR_tertile 3 vs 1 _= 2.01, 95% CI = 1.45 to 2.77) and among postmenopausal women (OR_quintile 5 vs 1 _= 2.19, 95% CI = 1.65 to 2.89). However, increasing absolute nondense area was associated with a decreased risk of breast cancer among premenopausal women (OR_tertile 3 vs 1 _= 0.51, 95% CI = 0.36 to 0.72) and among postmenopausal women (OR_quintile 5 vs 1 _= 0.46, 95% CI = 0.34 to 0.62). These associations changed minimally when we included both absolute dense area and absolute nondense area in the same statistical model. As expected, the percentage dense area was the strongest risk factor for breast cancer in both groups.

**Conclusions:**

Our results indicate that absolute dense area is independently and positively associated with breast cancer risk, whereas absolute nondense area is independently and inversely associated with breast cancer risk. Since adipose tissue is radiographically nondense, these results suggest that adipose breast tissue may have a protective role in breast carcinogenesis.

## Introduction

Mammographic density is one of the strongest predictors of breast cancer risk. Breast epithelium and stroma are radiographically dense and appear light on a mammogram, whereas adipose tissue is nondense and appears dark. Women with more than 75% of the total area on a mammogram occupied by dense area have a four- to sixfold greater risk of breast cancer compared to women with little or no dense area on a mammogram [1,2]. Although percentage dense area is the most commonly used measurement of mammographic density, absolute dense area is also associated with breast cancer risk. Most studies that have investigated both absolute and percentage dense area in relation to breast cancer risk have reported similar or stronger associations with percentage dense area [1,3-9].

The biological mechanisms underlying the association between mammographic density and breast cancer risk are unclear. It is plausible that mammographic density is related to breast cancer risk because it reflects the total amount of epithelium and stroma in the breasts [10]. The association between absolute dense area and breast cancer risk supports this view, since the dense area on a mammogram is a measure of mammary tissue. Percentage dense area, however, appears to be a better predictor of breast cancer risk. A possible explanation of this paradox is that the nondense area on a mammogram, which is part of the denominator in percentage dense area, is inversely related to breast cancer risk.

In this study, we separately investigated absolute dense area, absolute nondense area and percentage dense area in relation to breast cancer risk. The aim of the study was to better understand the aspects of the association between mammographic density and risk of breast cancer. *A priori *we expected there to be a positive association between absolute dense area and percentage dense area and risk of breast cancer. We had no *a priori *hypothesis regarding the association between nondense mammographic area and risk of breast cancer.

## Materials and methods

We conducted a nested case-control study on mammographic density and risk of breast cancer in the Nurses' Health Study (NHS) and Nurses' Health Study II (NHS II).

### The Nurses' Health Study

The NHS started in 1976, when 121,700 US registered nurses ages 30 to 55 years completed a questionnaire about their medical history, reproductive history and lifestyle habits [11]. Follow-up questionnaires have been mailed to the participants every two years since (response rate > 90% per questionnaire cycle). In 1989 and 1990, a total of 32,826 participants provided blood samples.

In the current study, we made use of an existing breast cancer case-control study nested within the subcohort of women who provided a blood sample and had no history of breast cancer at the time they provided the sample. During follow-up to 1 June 2004, there were 1,897 incident cases of breast cancer reported on the biennial questionnaire and confirmed by medical record review. These cases were matched to 3,165 controls on age, menopausal status, current use of postmenopausal hormones, month, time of day and fasting status at the time of blood collection. At the time of mammographic data collection, 1,612 cases and 2,857 controls were alive and were sent letters requesting their participation in the study. We attempted to obtain mammograms taken as close to the date of blood collection as possible from 1,504 cases and 2,512 controls who gave their permission to participate. We obtained mammograms from 1,446 cases and 2,406 controls. We excluded 37 cases and 35 controls because of poor-quality mammograms, 104 cases whose date of mammogram was after the date of the breast cancer diagnosis and 9 controls with discrepant information regarding menopausal status at the date of the mammogram and at the date of diagnosis/index date. In total, we had usable mammograms for 1,305 cases and 2,362 controls. The median time between mammography and blood draw was 11 months before blood collection (IQR = 46 months). The median time between mammography and breast cancer diagnosis was 4.6 years (IQR = 5.3 years).

### The Nurses' Health Study II

The NHS II started in 1989, when 116,686 US registered nurses ages 25 to 42 years completed a questionnaire about their medical history, reproductive history and lifestyle habits [11]. Follow-up questionnaires have been mailed to the participants every two years since (response rate approximately 90% per questionnaire cycle). From 1995 through 1999, a total of 29,611 participants provided blood samples.

As in the NHS, our current study was nested within the subcohort of women who had no history of breast cancer at the time they provided a blood sample. During follow-up to 1 June 2003, there were 318 incident cases of breast cancer reported on the biennial questionnaire and confirmed by medical record review. These cases were matched to 635 controls on age, menopausal status, current use of postmenopausal hormones, month, time of day and fasting status at the time of blood collection. At the time of mammographic data collection, 304 cases and 624 controls were alive and were sent letters requesting their participation. We attempted to obtain mammograms taken as close to the date of blood collection as possible from 274 cases and 519 controls who gave their permission to participate. We obtained mammograms from 253 cases and 502 controls. We excluded 13 cases and 15 controls because of poor-quality mammograms, 17 cases whose date of mammogram was after the date of the breast cancer diagnosis, and 1 case and 5 controls with discrepant information regarding menopausal status at the date of the mammogram and at the date of diagnosis/index date. In total, we had usable mammograms for 222 cases and 482 controls. The median time between mammography and blood draw was 3 months before blood collection (IQR = 18 months). The median time between mammography and breast cancer diagnosis was 2.1 years (IQR = 2.6 years).

### Mammographic density measurements

The detailed methods used for mammographic density measurement in this study have been published previously [12]. Briefly, the craniocaudal views of both breasts were digitized with a Lumisys 85 digital laser film scanner (Lumisys, Sunnyvale, CA, USA). We used Cumulus software (Canto Software Inc, San Francisco, CA, USA) for computer-assisted thresholding to measure absolute dense area, absolute nondense area (the total area minus the dense area) and percentage dense area (the dense area divided by the total area). For each batch of mammograms on which the observer measures density, a random sample of replicate mammograms were included as quality control samples to assess the reliability of the reader. The observer was blinded to the case-control and quality control status of the mammograms. For each batch, the within-person intraclass correlation coefficient has been at least 0.90. The average density parameters of both breasts were used for this analysis.

### Covariate information

We used the most recent information from the two biennial questionnaires preceding the date of the mammogram to obtain data on the following covariates: body mass index (BMI in kg/m^2^), current postmenopausal hormone use (PMH) and alcohol intake (g/day). Thus information on BMI, PMH use and alcohol intake was extracted from questionnaires returned by the participants 0 to 4 years before mammography. For the covariates age at menarche, menopausal status, parity, age at first childbirth, age at menopause and family history of breast cancer, we used the most recent information from any biennial questionnaire returned by the participant before the mammography.

### Statistical analysis

We combined the cases and controls from NHS and NHS II to increase the power of our study. We excluded women with unknown menopausal status at the time of mammogram (*n *= 287). After these exclusions, 464 cases and 998 controls with premenopausal mammograms and 960 cases and 1,662 controls with postmenopausal mammograms remained for analysis.

Among women with premenopausal mammograms, we divided the mammographic density measurements into tertiles based on the distribution among the controls. Given the larger sample size of postmenopausal women compared with premenopausal women, we were able to examine finer categories of the distribution among postmenopausal women. Among women with postmenopausal mammograms, the mammographic density measurements were divided into quintiles based on the distribution among the controls. All covariates except for alcohol consumption and PMH use had missing data for less than 2% of both cases and controls and were assigned the reference category or median value. Women with missing information on alcohol consumption (*n *= 232) or PMH use (*n *= 210) were assigned a missing indicator variable. As a sensitivity analysis, we restricted the analysis to women with no missing information on any covariates (420 cases and 917 controls with premenopausal mammograms and 806 cases and 1,432 controls with postmenopausal mammograms).

In the original nested case-control studies described above, controls were matched to cases on age and on other variables related to blood collection. For the current study, because we were unable to obtain mammograms for all eligible women, the original matching was not maintained. Therefore, we used unconditional logistic regression to calculate OR and 95% CI data and adjusted all models for age at mammography (continuous) and study (NHS or NHS II). The multivariate model was additionally adjusted for the following categorical and continuous variables using data reported closest in time to the mammography: BMI (< 20, 20 to < 22.5, 22.5 to < 25, 25 to < 27.5, 27.5 to < 30, 30 to < 32.5, 32.5 to < 35 and ≥ 35), age at menarche (< 12, 12 to 13 and ≥ 14 years), family history of breast cancer (yes or no), parity and age at first childbirth (nulliparous, < 3 children by age < 26 years, < 3 children by age ≥ 26 years, ≥ 3 children by age < 26 years and ≥ 3 children by age ≥ 26 years), alcohol use (0, < 5, 5 to < 15, ≥ 15 g/day or unknown), and, for women who were postmenopausal at the time of mammography, PMH use (yes, no or unknown) and age at menopause (continuous). We also adjusted for absolute dense area and absolute nondense area simultaneously in the multivariate model. We assessed the interaction between absolute dense area and nondense area with respect to breast cancer risk on the continuous scale. Absolute dense area, absolute nondense area and the cross-product of these factors were entered to the multivariate models as continuous variables, and we used the Wald test to assess the statistical significance of the interaction terms. We also investigated, on the continuous scale, whether there was an interaction between the different measurements of mammographic density and BMI.

Since premenopausal and postmenopausal breast cancer may have, in part, different etiologies, and because some women who were premenopausal at the time of mammography were postmenopausal at the time of breast cancer diagnosis, we conducted a sensitivity analysis in which we restricted that analysis to women who were premenopausal at the time of mammography and at the time of breast cancer diagnosis (263 cases and 586 controls). This study was approved by the Committee on the Use of Human Subjects in Research at Brigham and Women's Hospital. All participants in this study provided informed consent.

## Results

In this study, there were 464 breast cancer cases and 998 controls with premenopausal mammograms, as well as 960 breast cancer cases and 1,662 controls with postmenopausal mammograms. Among controls, premenopausal women with higher absolute dense area were younger at the time of mammography, were somewhat older at the time of menarche, were more often nulliparous, were older when they had their first child, had fewer children and were leaner (Table 1). Women with higher absolute nondense area tended to be younger at menarche, consumed less alcohol and had considerably higher BMI. Trends across categories of percentage dense area were largely similar to those for absolute dense area, although women with higher percentage dense area consumed more alcohol and had noticeably lower BMI. A similar descriptive analysis among postmenopausal controls is presented in Table 2. With increasing absolute dense area, women had fewer children, were somewhat older when they had their first child and more often used PMH. With increasing absolute nondense area, women had higher BMI, were younger when they had their first child and at the time of menopause, and consumed less alcohol. As among premenopausal women, percentage dense area largely reflected the results for absolute dense area. Notably, women with higher percentage dense area were younger at mammography, had lower BMI and more often used PMH. The correlations between absolute dense and nondense areas were 0.03 in premenopausal women and 0.03 in postmenopausal women.

**Table 1:** Age-adjusted (≤45, 46+ years) characteristics at premenopausal mammography among 998 control women without breast cancer according to tertiles of absolute dense area, absolute non-dense area, and percent dense area

	Tertiles of absolute dense area in cm^2^		
Characteristics	1 (< 42.5)	2 (< 79.3)	3 (< 300.1)
Number of controls	330	339	329
Age at mammography, years	48.7	47.3	45.8
Age at menarche, years	12.3	12.4	12.5
Family history of breast cancer, %	6.9	8.6	8.0
Nulliparous at the time of mammography, %	7.2	7.6	13.9
Number of children at mammography^1^	2.7	2.6	2.4
Age at first birth, years^1^	25.1	25.7	26.1
Body mass index at mammography, kg/m^2^	26.6	24.7	24.7
Alcohol use at mammography, g/day	4.1	5.5	4.6
Total area on mammogram, cm^2^	164	177	253
Absolute non-dense area on mammogram, cm^2^	139	117	131
	Tertiles of absolute non-dense area in cm^2^		
Characteristics	1 (< 69.5)	2 (< 145.1)	3 (< 482.6)

Number of controls	330	339	329
Age at mammography, years	47.3	47.4	47.2
Age at menarche, years	12.5	12.5	12.2
Family history of breast cancer, %	6.6	8.6	7.3
Nulliparous at the time of mammography, %	11.7	8.3	8.8
Number of children at mammography^1^	2.5	2.6	2.6
Age at first birth, years^1^	25.6	25.8	25.5
Body mass index at mammography, kg/m^2^	22.4	24.6	28.7
Alcohol use at mammography, g/day	5.8	4.7	3.9
Total area on mammogram, cm^2^	105	181	304
Absolute dense area on mammogram, cm^2^	62	76	70
	Tertiles of percent dense area		
Characteristics	1 (< 27.8)	2 (< 49.9)	3 (< 90.4)

Number of controls	330	339	329
Age at mammography, years	48.2	47.4	46.4
Age at menarche, years	12.1	12.5	12.6
Family history of breast cancer, %	6.5	9.2	7.2
Nulliparous at the time of mammography, %	7.1	9.3	12.5
Number of children at mammography^1^	2.8	2.6	2.4
Age at first birth, years^1^	25.0	26.2	25.7
Body mass index at mammography, kg/m^2^	28.5	24.5	22.7
Alcohol use at mammography, g/day	4.1	4.9	5.5
Total area on mammogram, cm^2^	238	194	156
Absolute dense area on mammogram, cm^2^	35	75	96
Absolute non-dense area on mammogram, cm^2^	203	119	59

^1) ^Among parous women.

**Table 2:** Age-adjusted (< 55, 55-59, 60-64, 65+ years) characteristics at postmenopausal mammography among 1,662 control women without breast cancer according to quintiles of absolute dense area, absolute non-dense area, and percent dense area

	Quintiles of absolute dense area in cm^2^				
Characteristics	1 (< 14.4)	2 (< 26.6)	3 (< 39.8)	4 (< 64.3)	5 (< 302.6)
Number of controls	333	332	333	332	332
Age at mammography, years	62.2	61.4	62.0	60.6	60.4
Age at menopause, years	49.2	49.5	49.8	49.0	49.0
Age at menarche, years	12.6	12.7	12.6	12.6	12.6
Family history of breast cancer, %	11.6	12.0	14.0	13.0	13.2
Nulliparous at the time of mammography, %	4.0	5.5	5.2	8.0	10.2
Number of children at mammography^1^	3.6	3.6	3.4	3.2	3.2
Age at first child, years^1^	25.1	25.1	25.2	25.4	25.4
Body mass index at mammography, kg/m^2^	28.2	26.2	25.4	25.1	25.5
Alcohol use at mammography, g/day	5.9	5.7	4.7	5.9	4.9
Current hormonal use, %	30.3	34.6	34.8	42.4	56.1
Total area on mammogram, cm^2^	169	169	182	216	268
Absolute non-dense area on mammogram, cm^2^	162	148	149	164	171
	Quintiles of absolute non-dense area in cm^2^				
Characteristics	1 (< 76.2)	2 (< 115.6)	3 (< 163.9)	4 (< 231.4)	5 (< 538.8)

Number of controls	333	332	333	332	332
Age at mammography, years	60.7	60.4	61.6	62.1	61.9
Age at menopause, years	49.6	49.5	49.2	49.2	48.9
Age at menarche, years	12.8	12.6	12.6	12.5	12.4
Family history of breast cancer, %	12.1	14.2	11.8	13.0	12.6
Nulliparous at the time of mammography, %	8.3	8.4	5.3	5.1	6.4
Number of children at mammography^1^	3.4	3.5	3.4	3.4	3.3
Age at first child, years^1^	25.5	25.4	25.2	25.2	24.9
Body mass index at mammography, kg/m^2^	22.4	24.7	25.9	27.5	30.1
Alcohol use at mammography, g/day	6.9	6.0	5.6	5.2	3.2
Current hormonal use, %	47.6	39.9	33.8	38.6	36.1
Total area on mammogram, cm^2^	93	135	179	241	355
Absolute dense area on mammogram, cm^2^	41	39	40	45	46
	Quintiles of percent dense area				
Characteristics	1 (< 8.1)	2 (< 15.7)	3 (< 24.1)	4 (< 37.1)	5 (< 86.5)

Number of controls	334	331	333	332	332
Age at mammography, years	62.2	62.0	61.8	60.8	59.7
Age at menopause, years	49.1	49.7	49.3	49.1	49.0
Age at menarche, years	12.6	12.5	12.5	12.7	12.8
Family history of breast cancer, %	10.7	14.3	12.0	12.8	13.9
Nulliparous at the time of mammography, %	6.2	2.7	5.5	8.0	11.4
Number of children at mammography^1^	3.6	3.5	3.4	3.2	3.1
Age at first child, years^1^	25.2	24.8	25.1	25.4	25.8
Body mass index at mammography, kg/m^2^	29.9	26.9	25.7	24.4	23.4
Alcohol use at mammography, g/day	5.2	4.3	5.4	6.0	6.1
Current hormonal use, %	27.1	32.7	36.4	47.3	54.1
Total area on mammogram, cm^2^	231	223	210	182	154
Absolute dense area on mammogram, cm^2^	10	27	41	55	76
Non-dense breast area on mammogram, cm^2^	221	196	169	127	78

^1) ^Among parous women.

Increasing absolute dense area was associated with a greater risk of breast cancer after we adjusted for potential confounding factors among premenopausal women (OR_tertile 3 vs 1 _= 2.01, 95% CI = 1.45 to 2.77) and postmenopausal women (OR_quintile 5 vs 1 _= 2.19; 95% CI = 1.65 to 2.89) (Tables 3 and 4). In contrast, increasing absolute nondense area was associated with a decreased risk of breast cancer among premenopausal women (OR_tertile 3 vs 1 _= 0.51, 95% CI = 0.36 to 0.72) and postmenopausal women (OR_quintile 5 vs 1 _= 0.46; 95% CI = 0.34 to 0.62). When we additionally mutually adjusted for absolute dense area and absolute nondense area, the results were generally similar. Among both pre- and postmenopausal women, the percentage dense area was most strongly associated with breast cancer. We found no significant interactions between absolute dense area, absolute nondense area, or percentage dense area and BMI among premenopausal or postmenopausal women (all *P*-values > 0.05).

**Table 3:** Odds ratios for breast cancer according to tertiles of different premenopausal mammographic characteristics among 464 cases and 998 controls

	Odds Ratios and 95% Confidence Intervals							
Mammographic characteristics	Cases	Controls	Model 1^1^		Model 2^2^		Model 3^3^	
Absolute dense area, tertiles								
1	113	330	1.00		1.00		1.00	
2	156	339	1.43	(1.07-1.92)	1.40	(1.04-1.88)	1.39	(1.03-1.87)
3	195	329	2.07	(1.52-2.84)	2.01	(1.45-2.77)	2.05	(1.48-2.84)
Absolute non-dense area, tertiles								
1	192	330	1.00		1.00		1.00	
2	153	339	0.77	(0.59-1.01)	0.72	(0.54-0.95)	0.69	(0.52-0.92)
3	119	329	0.62	(0.47-0.83)	0.51	(0.36-0.72)	0.50	(0.35-0.71)
Percent dense area, tertiles								
1	95	330	1.00		1.00		N/A	
2	167	339	1.75	(1.30-2.36)	1.95	(1.41-2.69)		
3	202	329	2.18	(1.62-2.92)	2.72	(1.93-3.83)		

^1) ^Adjusted for age at mammography (continuous), study (Nurses' Health Study, Nurses' Health Study II).

^2) ^Adjusted for age at mammography (continuous), study (Nurses' Health Study, Nurses' Health Study II), age at menarche (< 12, 12-13, ≥ 14), family history of breast cancer (yes, no), parity + age at first birth (nulliparous, < 3 children age < 26, < 3 children age ≥ 26, ≥ 3 children age < 26, ≥ 3 children age ≥ 26), body mass index (< 20, 20-< 22.5, 22.5-< 25, 25-< 27.5, 27.5-< 30, 30-< 32.5, 32.5-< 35, ≥ 35 kg/m^2^), and alcohol use (0, < 5, 5-< 15, ≥ 15 g/day, unknown).

^3) ^Adjusted for covariates in model 2, plus absolute non-dense or absolute dense area (tertiles), as appropriate.

**Table 4:** Odds ratios for breast cancer according to quintiles of different postmenopausal mammographic characteristics among 960 cases and 1,662 controls

	Odds Ratios and 95% Confidence Intervals							
Mammographic characteristics	Cases	Controls	Model 1^1^		Model 2^2^		Model 3^3^	
Absolute dense area, quintiles								
1	117	333	1.00		1.00		1.00	
2	167	332	1.42	(1.08-1.89)	1.44	(1.08-1.92)	1.39	(1.04-1.86)
3	167	333	1.43	(1.08-1.89)	1.44	(1.08-1.92)	1.42	(1.06-1.90)
4	240	332	2.07	(1.58-2.71)	1.99	(1.50-2.62)	2.04	(1.53-2.70)
5	269	332	2.40	(1.83-3.13)	2.19	(1.65-2.89)	2.30	(1.74-3.06)
Absolute non-dense area, quintiles								
1	271	333	1.00		1.00		1.00	
2	177	332	0.66	(0.52-0.84)	0.60	(0.47-0.78)	0.62	(0.47-0.80)
3	186	333	0.70	(0.55-0.89)	0.63	(0.48-0.82)	0.62	(0.48-0.82)
4	178	332	0.68	(0.53-0.86)	0.58	(0.44-0.76)	0.55	(0.41-0.72)
5	148	332	0.56	(0.44-0.73)	0.46	(0.34-0.62)	0.41	(0.30-0.56)
Percent dense area, quintiles								
1	109	334	1.00		1.00		N/A	
2	161	331	1.49	(1.12-1.98)	1.56	(1.16-2.10)		
3	153	333	1.42	(1.06-1.89)	1.57	(1.16-2.13)		
4	231	332	2.13	(1.62-2.81)	2.44	(1.81-3.30)		
5	306	332	2.83	(2.17-3.71)	3.28	(2.41-4.45)		

^1) ^Adjusted for age at mammography (continuous), study (Nurses' Health Study, Nurses' Health Study II).

^2) ^Adjusted for age at mammography (continuous), study (Nurses' Health Study, Nurses' Health Study II), age at menarche (< 12, 12-13, ≥ 14), family history of breast cancer (yes, no), parity + age at first birth (nulliparous, < 3 children age < 26, < 3 children age ≥ 26, ≥ 3 children age < 26, ≥ 3 children age ≥ 26), body mass index (< 20, 20-< 22.5, 22.5-< 25, 25-< 27.5, 27.5-< 30, 30-< 32.5, 32.5-< 35, ≥ 35 kg/m^2^), alcohol use (0, < 5, 5-< 15, ≥ 15 g/day, unknown), age at menopause (continuous), and current hormonal therapy (yes, no, unknown).

^3) ^Adjusted for covariates in model 2, plus absolute non-dense or absolute dense area (quintiles), as appropriate.

Table 5 and 6 show risk estimates for breast cancer by cross-classified categories of absolute dense area and absolute nondense area. In both pre- and postmenopausal women, the risk of breast cancer increased with higher absolute dense area within each category of absolute nondense area, and, conversely, the risk decreased with higher absolute nondense area within each category of absolute dense area (*P *= 0.26 for interaction for premenopausal mammographic measurements, and *P *= 0.97 for interaction for postmenopausal mammographic measurements).

**Table 5:** Multivariate^1 ^odds ratios (ORs) and 95% Confidence Intervals (CIs) for breast cancer by tertiles of absolute dense area and absolute non-dense area on a premenopausal mammogram

	Tertiles non-dense area		
	1 OR (95% CI)	2 OR (95% CI)	3 OR (95% CI)
Tertiles dense area			
1	2.40 (1.30-4.46)	2.13 (1.15-3.93)	1.00
cases/controls	51/122	38/91	24/117
2	3.44 (1.91-6.18)	2.28 (1.26-4.14)	1.76 (0.96-3.22)
cases/controls	76/131	46/111	34/97
3	5.10 (2.78-9.37)	3.16 (1.78-5.63)	2.78 (1.57-4.92)
cases/controls	65/77	69/137	61/115

P interaction = 0.26

^1) ^Adjusted for age at mammography (continuous), study (Nurses' Health Study, Nurses' Health Study II), age at menarche (< 12, 12-13, ≥ 14), family history of breast cancer (yes, no), parity + age at first birth (nulliparous, < 3 children age < 26, < 3 children age ≥ 26, ≥ 3 children age < 26, ≥ 3 children age ≥ 26), body mass index (< 20, 20-< 22.5, 22.5-< 25, 25-< 27.5, 27.5-< 30, 30-< 32.5, 32.5-< 35, ≥ 35 kg/m^2^), and alcohol use (0, < 5, 5-< 15, ≥ 15 g/day, unknown).

**Table 6:** Multivariate^1 ^odds ratios (ORs) and 95% Confidence Intervals (CIs) for breast cancer by quintiles of absolute dense area and absolute non-dense area on a postmenopausal mammogram

	Quintiles non-dense area				
	1 OR (95% CI)	2 OR (95% CI)	3 OR (95% CI)	4 OR (95% CI)	5 OR (95% CI)
Quintiles dense area					
1	2.92 (1.23-6.93)	1.39 (0.66-2.90)	1.73 (0.86-3.50)	1.04 (0.50-2.16)	1.00
cases/controls	16/29	26/80	35/85	24/83	16/56
2	3.75 (1.89-7.45)	1.98 (0.94-4.17)	1.58 (0.76-3.29)	2.05 (0.95-4.43)	1.42 (0.68-2.96)
cases/controls	65/91	27/62	27/74	23/43	25/62
3	3.68 (1.84-7.36)	1.84 (0.89-3.79)	1.58 (0.73-3.42)	2.05 (0.99-4.25)	1.73 (0.83-3.62)
cases/controls	59/82	31/77	21/56	30/62	26/56
4	4.82 (2.44-9.53)	3.30 (1.60-6.80)	3.01 (1.46-6.20)	2.91 (1.45-5.85)	1.80 (0.90-3.60)
cases/controls	80/76	41/53	37/55	44/65	38/83
5	4.29 (2.10-8.76)	3.83 (1.90-7.75)	4.36 (2.20-8.64)	2.88 (1.46-5.66)	2.15 (1.08-4.26)
cases/controls	51/55	52/60	66/63	57/79	43/75

P interaction = 0.97

^1) ^Adjusted for age at mammography (continuous), study (Nurses' Health Study, Nurses' Health Study II), age at menarche (< 12, 12-13, ≥ 14), family history of breast cancer (yes, no), parity + age at first birth (nulliparous, < 3 children age < 26, < 3 children age ≥ 26, ≥ 3 children age < 26, ≥ 3 children age ≥ 26), body mass index (< 20, 20-< 22.5, 22.5-< 25, 25-< 27.5, 27.5-< 30, 30-< 32.5, 32.5-< 35, ≥ 35 kg/m^2^), alcohol use (0, < 5, 5-< 15, ≥ 15 g/day, unknown), age at menopause (continuous), and current hormonal therapy (yes, no, unknown).

In a sensitivity analysis restricted to women who were premenopausal both at the time of mammography and at the time of breast cancer diagnosis, the results were generally similar (Additional file 1, Table S1). Also, results from the sensitivity analysis restricted to cases and controls with information on all covariates were quantitatively similar to the results from the primary analysis (data not shown).

## Discussion

We found that higher absolute dense area on a mammogram was associated with an increased risk of breast cancer, whereas higher absolute nondense area was associated with a decreased risk. When we adjusted for both of these factors simultaneously, the associations remained. Our results suggest that absolute dense area is independently and positively associated with breast cancer risk, whereas absolute nondense area is independently and inversely associated with breast cancer risk.

Two previous studies have reported the association between absolute nondense area and the risk of breast cancer. The UK Guernsey prospective studies (111 cases of breast cancer) found that women in the highest versus lowest quartile of absolute nondense area had a hazard ratio of 0.56 (95% CI = 0.29 to 1.11; *P *for trend = 0.18) for breast cancer, but reported no risk estimates for absolute nondense area adjusted for absolute dense area [7]. Stone and colleagues [6], in a case-control study of 634 cases and 1,880 controls, found that women in the highest versus lowest quintile of absolute nondense area had an OR of 0.80 (95% CI = 0.60 to 1.08; *P *for trend = 0.02) for breast cancer. After adjustment for absolute dense area, the OR changed to 1.08 (95% CI = 0.79 to 1.49; *P *for trend = 0.7). Although both of these studies suggest an inverse association between nondense area and breast cancer risk, it is unclear why adjustment for dense area in the Stone *et al*. study had a different effect than in the current study. Both studies used the same computer-assisted thresholding techniques to measure mammographic density; however, Stone and colleagues used the mediolateral oblique mammogram view, whereas we used the craniocaudal view. Although the mammogram view may affect the mammographic density measurements and thus their ability to predict breast cancer risk, it seems unlikely to explain our different study findings [6,13]. Taken together, the unexplained differences in study findings underscore the importance of investigating the association between the nondense area on a mammogram and the risk of breast cancer in additional independent cohorts.

Prior findings published by Stuedal and colleagues [14] lend indirect to support to the notion that nondense mammographic area is inversely associated with breast cancer risk. Stuedal and colleagues examined if the association between mammographic density and breast cancer risk differs according to the size of the breasts. They used the total breast area on a mammogram as a proxy for breast size and found that the association between absolute dense area and breast cancer risk was significantly lower in larger breasts [14]. For percentage dense area, the results were similar, although weaker and nonsignificant. Their results suggest that absolute dense area, and possibly percentage dense area, is a weaker predictor of breast cancer risk in larger breasts. Stuedal and colleagues put forth several possible explanations for their findings, including measurement error, a higher proportion of dense tissue in the largest breasts may be less correlated with the number of epithelial cells at risk, and fat in the breast having a potential protective effect on breast cancer risk.

Previous research has led to the hypotheses that mammographic density reflects the proliferation of epithelial and stromal cells [15,16], the number of mammary stem cells at risk of developing breast cancer [10] and/or the cumulative exposure of the breast to different mitogens and mutagens [16]. If these hypotheses are correct, absolute dense volume should be a better predictor of breast cancer risk than percentage dense area or percentage dense volume. Boyd and colleagues used two different novel techniques to estimate absolute and percentage dense volume on the basis of mammograms [3,17]. In these studies, absolute dense volume was not found to be a stronger risk factor for breast cancer than percentage dense area or volume, suggesting that percentage dense area or volume contains information about breast cancer risk that goes beyond the total amount of epithelium and stroma in the breasts.

Several different factors or mechanisms can explain the inverse association between absolute nondense area and breast cancer risk that we found. Absolute nondense area may be a marker of some other factors that in turn are true protective factors for both premenopausal and postmenopausal breast cancer. Although BMI is the strongest confounder of this association, adjusting for BMI did not explain but a small fraction of the association. In addition, we controlled for several other potential confounding factors, and adjusting for these factors did not materially alter the results, although some residual confounding cannot be excluded.

Our results support the view that breast fat could have a protective effect on breast cancer risk [14]. Little is known about the role of breast adipose tissue in breast carcinogenesis. On the basis of previous literature, however, it appears more likely that higher amounts of adipose breast tissue would increase rather than decrease the risk of breast cancer [18]. Adipose tissue is an important source of estrogens, and higher levels of estrogens, especially locally, would presumably increase breast cancer development and progression. In addition, increased adiposity is associated with increased levels of potentially cancer-promoting adipokines, such as leptin, and decreased levels of potential anticancer adipokines, such as adiponectin [19]. A possible explanation of our findings, in addition to adipose tissue having a potential protective effect *per se*, is that the dense area and the nondense area on a mammogram reflect the extent of conversion of the breast tissue into either dense tissue that is associated with breast cancer risk or nondense tissue that is not. Lobular involution is inversely associated with breast cancer risk. Ghosh and colleagues [20] reported an inverse association between involution and percentage dense area; specifically, they found no association between involution and absolute dense area, but a positive association between involution and absolute nondense area.

A limitation of this study is that we were unable to collect mammograms from all eligible women. However, we have no reason to suspect that women with missing mammograms are systematically different from women for whom we were able to collect mammograms. In addition, our findings regarding absolute dense area and percentage dense area in relation to breast cancer risk are in line with those of numerous previous studies, indicating that our measurements of mammographic density, including absolute nondense area, are valid. Another limitation of this study is that information on some covariates that can change substantially over a short period of time, notably BMI, PMH use and alcohol consumption, was retrieved from questionnaires returned by the participants up to four years before mammography. This could have led to misclassification of the covariates and thus to residual confounding.

## Conclusions

Our results indicate that absolute dense area is independently and positively associated with breast cancer risk, whereas absolute nondense area is independently and inversely associated with breast cancer risk. We propose that the nondense area on a mammogram should be investigated as a distinct factor when studying the association between mammographic patterns and risk of breast cancer. Furthermore, our results suggest that adipose breast tissue may have an important protective role in breast carcinogenesis.

## Abbreviations

BMI: body mass index; CI: confidence interval; IQR: inter-quartile range; NHS II: Nurses' Health Study II; NHS: Nurses' Health Study; OR: odds ratio; PMH: postmenopausal hormone.

## Competing interests

The authors declare that they have no competing interests.

## Authors' contributions

RMT designed the case-control study used for this analysis, came up with the analytical approach used for this analysis and drafted the manuscript. AP came up with the analytical approach used for this analysis, conducted the data analysis and drafted the manuscript. SEH and WCW designed the case-control study used for this analysis. PL and DT came up with the analytical approach used for this analysis. All authors interpreted the results. All authors edited the manuscript and approved the final manuscript submitted for publication.

## Supplementary Material

Additional file 1 **Table S1: Odds ratios for breast cancer among women who were premenopausal at the time of mammography and at the time of breast cancer diagnosis according to tertiles of different mammographic characteristics among 263 cases and 586 controls**. Click here for file
