# Supplementary material for: Nondense mammographic area and risk of breast cancer
Source: Breast Cancer Res. 2011 Oct 21;13(5):R100. doi: 10.1186/bcr3041 (PMC3262213; doi:10.1186/bcr3041)
Supplement: Additional file 1 — Table S1: Odds ratios for breast cancer among women who were premenopausal at the time of mammography and at the time of breast cancer diagnosis according to tertiles of different mammographic characteristics among 263 cases and 586 controls. [file bcr3041-S1.DOC]

| Additional File 1 Table S1. Odds ratios for breast cancer among women who were premenopausal at the time of mammography and at the time of breast cancer diagnosis according to tertiles of different mammographic characteristics among 263 cases and 586 controls. | | | | | | | | |
| --- | --- | --- | --- | --- | --- | --- | --- | --- |
|  | Odds Ratios and 95% Confidence Intervals | | | | | | | |
| Mammographic characteristics | Cases | Controls | Model 1^1^ | | Model 2^2^ | | Model 3^3^ | |
| Absolute dense area, tertiles |  |  |  |  |  |  |  |  |
| 1 | 60 | 194 | 1.00 |  | 1.00 |  | 1.00 |  |
| 2 | 85 | 199 | 1.63 | (1.08-2.47) | 1.57 | (1.02-2.41) | 1.47 | (0.95-2.27) |
| 3 | 118 | 193 | 2.61 | (1.67-4.08) | 2.48 | (1.55-3.96) | 2.34 | (1.46-3.78) |
| Absolute non-dense area, tertiles |  |  |  |  |  |  |  |  |
| 1 | 112 | 194 | 1.00 |  | 1.00 |  | 1.00 |  |
| 2 | 87 | 199 | 0.73 | (0.51-1.04) | 0.67 | (0.46-0.99) | 0.67 | (0.45-0.99) |
| 3 | 64 | 193 | 0.55 | (0.37-0.81) | 0.37 | (0.22-0.61) | 0.39 | (0.24-0.65) |
| Percent dense area, tertiles |  |  |  |  |  |  |  |  |
| 1 | 53 | 194 | 1.00 |  | 1.00 |  | N/A |  |
| 2 | 95 | 200 | 1.74 | (1.17-2.58) | 1.93 | (1.24-3.00) |  |  |
| 3 | 115 | 192 | 2.16 | (1.46-3.17) | 2.82 | (1.76-4.51) |  |  |
| ^1)^ Adjusted for age at mammography (continuous), study (Nurses’ Health Study, Nurses’ Health Study II).  ^2)^ Adjusted for age at mammography (continuous), study ( Nurses’ Health Study, Nurses’ Health Study II), age at menarche (<12, 12-13, ≥14), family history of breast cancer (yes, no), parity + age at first birth (nulliparous, <3 children age <26, <3 children age ≥26, ≥3 children age <26, ≥3 children age ≥26), body mass index (<20, 20-<22.5, 22.5-<25, 25-<27.5, 27.5-<30, 30-<32.5, 32.5-<35, ≥35 kg/m^2^), and alcohol use (0, <5, 5-<15, ≥15 g/day, unknown).  ^3)^ Adjusted for covariates in model 2, plus absolute non-dense or absolute dense area (tertiles), as appropriate. | | | | | | | | |
